# Comparative transcriptomics of porcine liver-resident CD8α^dim^, liver CD8α^high^ and circulating blood CD8α^high^ NK cells reveals an intermediate phenotype of liver CD8α^high^ NK cells

**DOI:** 10.3389/fimmu.2023.1219078

**Published:** 2023-08-18

**Authors:** Leen Hermans, Sofie Denaeghel, Robert J. J. Jansens, Steffi De Pelsmaeker, Filip Van Nieuwerburgh, Dieter Deforce, Everardo Hegewisch-Solloa, Emily M. Mace, Eric Cox, Bert Devriendt, Herman W. Favoreel

**Affiliations:** ^1^ Department of Translational Physiology, Infectiology and Public Health, Faculty of Veterinary Medicine, Ghent University, Merelbeke, Belgium; ^2^ Department of Pharmacology, Weill Medical College, Cornell University, New York, NY, United States; ^3^ Faculty of Pharmaceutical Sciences, NXTGNT, Ghent University, Ghent, Belgium; ^4^ Department of Pediatrics, Vagelos College of Physicians and Surgeons, Columbia University Irving Medical Center, New York, NY, United States

**Keywords:** liver-resident, NK cells, transcriptome, pig, plasticity

## Abstract

Liver-resident NK (lrNK) cells have been studied in humans as well as in mice. Unfortunately, important differences have been observed between murine and human lrNK cells, complicating the extrapolation of data obtained in mice to man. We previously described two NK cell subsets in the porcine liver: A CD8α^high^ subset, with a phenotype much like conventional CD8α^high^ NK cells found in the peripheral blood, and a specific liver-resident CD8α^dim^ subset which phenotypically strongly resembles human lrNK cells. These data suggest that the pig might be an attractive model for studying lrNK cell biology. In the current study, we used RNA-seq to compare the transcriptome of three porcine NK cell populations: Conventional CD8α^high^ NK cells from peripheral blood (cNK cells), CD8α^high^ NK cells isolated from the liver, and the liver-specific CD8α^dim^ NK cells. We found that highly expressed transcripts in the CD8α^dim^ lrNK cell population mainly include genes associated with the (adaptive) immune response, whereas transcripts associated with cell migration and extravasation are much less expressed in this subset compared to cNK cells. Overall, our data indicate that CD8α^dim^ lrNK cells show an immature and anti-inflammatory phenotype. Interestingly, we also observed that the CD8α^high^ NK cell population that is present in the liver appears to represent a population with an intermediate phenotype. Indeed, while the transcriptome of these cells largely overlaps with that of cNK cells, they also express transcripts associated with liver residency, in particular *CXCR6*. The current, in-depth characterization of the transcriptome of porcine liver NK cell populations provides a basis to use the pig model for research into liver-resident NK cells.

## Introduction

The functional and phenotypical characteristics of peripheral blood natural killer (NK) cells, also referred to as conventional NK (cNK) cells, have been extensively studied since their discovery in the 1970s (1). They were found to predominantly kill virus-infected and malignant cells and produce large amounts of cytokines, particularly IFN-γ, upon stimulation (2). More recently, NK cell populations in non-lymphoid tissues, such as the liver, have been studied and characterized in more detail (3, 4). Liver-resident (lr) NK cells were first described as ‘pit cells’ in the rat liver in 1976 by electron microscopy (5). However, interest in this population was sparked particularly after O’Leary et al. discovered that an intra-hepatic NK cell population in mice could mediate long-lived and antigen-specific adaptive recall responses (6). This led to a better characterization of lrNK cell subsets in mice (7) and humans (8, 9).

Interestingly, lrNK cells are present at a remarkably high frequency as they make up 30-50% of all intrahepatic lymphocytes, compared to cNK cells representing only 5-15% of lymphocytes in the peripheral blood (10, 11). The lrNK population in humans and mice resides primarily in liver sinusoids and interacts with sinusoidal endothelial cells and Kupffer cells, the liver-resident macrophages (4, 12). However, their exact function in the liver is not yet fully understood (13). In mice, lrNK cells are CD49a-positive and express CXCR6 and TNF-related apoptosis-inducing ligand (TRAIL) (14–16). They express high levels of the T-box transcription factor T-bet and low levels of Eomesodermin (Eomes) (7). In contrast, the CD49a^+^ intrahepatic NK cell population in human represents only a very minor and variable subset of the total lrNK cell population (8). The majority of lrNK cells in human are defined as CD56^bright^CD49a^-^CD49e^-^CXCR6^+^CD69^+^ and show an opposite expression of the T-box transcription factors T-bet and Eomes (Eomes^high^T-bet^low^) compared to that in murine lrNK cells (9, 17, 18). Also in terms of (potential) plasticity between circulating cNK cells and lrNK cells, there appear to be differences between human and mice. In human, there are indications for such plasticity, e.g. circulating Eomes^low^ NK cells have been reported to upregulate Eomes expression under the influence of cytokines that are highly expressed in the liver (such as transforming growth factor β (TGF-β) and interleukin (IL)-15) (17). In mice, on the other hand, cNK and lrNK cell populations were shown to arise from separate lineages with only a limited degree of plasticity (19).

The abundant differences between human and mice lrNK cells (20) complicate extrapolation of data generated on murine lrNK cells to man. Interestingly, we recently identified two subpopulations of NK cells in the porcine liver (21). We described a CD8α^high^ subpopulation, with a phenotype much like conventional CD8α^high^ NK cells found in the peripheral blood, and an abundant liver-resident CD8α^dim^ subpopulation, which strongly resembles human lrNK cells (21). Indeed, like their human counterparts, porcine CD8α^dim^ lrNK cells are Eomes^high^T-bet^low^CXCR6^+^CD49e^-^ and there are indications for partial plasticity between porcine cNK cells and lrNK cells (21).

In the current study, we compared the transcriptome of porcine conventional liver CD8α^high^ NK cells, porcine liver-resident CD8α^dim^ NK cells, and porcine peripheral blood cNK cells using RNA-seq, to gain new insights into the potential biological functions of these still largely enigmatic lrNK cells. The in-depth characterization of porcine liver NK cell populations reported here, supports the future use of the pig as an animal model to study lrNK cells, which are difficult to obtain in human. These data form a basis to further characterize the biology and functions of porcine (and human) lrNK cells and possibly target these cells in future vaccination strategies and/or novel therapies against liver-associated diseases.

## Materials and methods

### Isolation of porcine blood NK cells

Heparinized blood samples (50 units/ml blood, LEO Pharma, Lier, Belgium) were obtained from the external jugular vein of pigs (11–23 weeks old) that were kept as blood donors at the Faculty of Veterinary Medicine, Merelbeke, Belgium. The blood sampling and euthanasia procedures were approved by the Ethical Committee of the Faculty of Veterinary Medicine (EC2017/121). Euthanasia was performed using Euthanimal 20% (natriumpentobarbital 20%, 60mg/2.5kg, IV). Peripheral blood NK cells used for RNA sequencing were isolated from the blood of five six-months-old pigs. Upon blood sampling, the animals were euthanized and the liver was collected to obtain liver NK cells (see section 2.2). NK cells used to perform plasticity experiments were obtained from other animals (11-23 weeks old).

Peripheral blood mononuclear cells (PBMC) were isolated from whole blood by lymphoprep (catalog n. 07851, Axis-Shield, Dundee, UK) density centrifugation. Red blood cells were lysed by a 10 min osmotic shock at room temperature by incubating the collected buffy coat in lysis buffer (composed of 90% NH_4_Cl 0.83% (w/v) and 10% TRIS 2.06% (w/v) in distilled water at a final pH of 7.2). After a final washing step, PBMC were resuspended in 1 ml phosphate-buffered saline (PBS)- ethylene diamine tetraacetic acid (EDTA) buffer and counted. Afterwards, primary porcine NK cells (CD3^−^CD172a^−^CD8α^+^) were isolated from porcine PBMC as described before (22) by magnetic-activated cell sorting (MACS) depletion of CD3^+^ and CD172a^+^ cells (Miltenyi, Bergisch Gladbach, Germany) followed by fluorescence-activated cell sorting (FACS) purification using a BD FACSMelody Cell Sorter (BD Biosciences, Franklin Lakes, NJ, USA) and monoclonal antibodies against porcine CD172a (IgG1, clone 74-22-15a), CD3 (IgG1, clone PPT3), and CD8α (IgG2a, clone 11/295/33) (23, 24). Secondary antibodies used were goat α-mouse IgG2a-AF647 (catalog n. A21241, Invitrogen, Carlsbad, CA, USA) and goat α-mouse IgG1-PE (catalog n. P21129, Invitrogen, Carlsbad, CA, USA). This resulted in a 98.42% ± 0.14% pure population of CD3^−^CD172a^−^CD8α^high^ blood NK cell population (**Supplementary Figures 1, 2**). In pigs, NK cells are typically identified based on aCD3^−^CD172a^−^CD8+ phenotype, since porcine NK cells do not express CD56 (24). This is in contrast to human NK cells, but similar to what is described in mice and non-human primates such as cynomolgus and rhesus macaques (23). Whether porcine NK cells express NKG2A or NKG2C on their cell surface is unknown and (cross-reactive) antibodies are currently lacking, preventing to use these molecules as NK cell markers in pig.

### Isolation of porcine liver NK cells

Blood was collected from five six-months-old pig (see section 2.1), after which the animals were euthanized and the liver was dissected and flushed to obtain liver NK cells from the same pig at the same age for RNA sequencing analysis. Porcine liver NK cells were isolated as described before (21). In short, the portal vein was perfused with 5 l ice-cold PBS, after which a further ice-cold 700 ml PBS was used for a final flush that was collected in different 50 ml centrifuge tubes, followed by concentration of the perfusate by centrifugation. The resulting cell pellet was resuspended in PBS (room temperature), followed by the same protocol described above for primary porcine blood NK cell isolation. FACS purification resulted in a 96.87% ± 0.99% pure population of porcine CD3^−^CD172a^−^CD8α^high^ liver NK cells and in a 95.14% ± 1.25% pure population of porcine CD3^−^CD172a^−^CD8α^dim^ liver NK cells (**Supplementary Figure 1**). Within the liver lymphocyte population, the CD8α^high^ NK cells represented 17.6 ± 8.2% of all cells, whereas the CD8α^dim^ NK cells represented 50.4 ± 16.3% of all cells. After sorting, the post-sort analysis was supplemented with additional control stainings for Tbet and Eomes, as these allow to carefully discriminate the two liver NK cell subpopulations (21) (**Supplementary Figure 2**).

### Immunofluorescence staining

After dissection and flushing of the liver, liver tissue samples were collected. The samples were embedded in 2% Methocel^®^ MC (catalog n. 9004-67-5, Fluka, Buchs, Switserland), snap-frozen in liquid nitrogen, and stored at -80°C until use. Cryosections (5 µm) were cut with a cryotome (Leica CM3050 S), placed on aminopropyltriethoxysilane (APES)-coated glass slides (catalog n. A3648, Sigma-Aldrich, Saint Louis, MO, USA) and fixed in methanol for 30 minutes at -20°C. The slides were dried and stored at -20° until use. Tissue slides were washed 3x 5 minutes with PBS and blocked with PBS + 10% goat serum for 30 minutes in a humid cell at 37°C. Primary antibodies (**Table 1**) were diluted in PBS and incubated for 1 h in a humid cell at 37°C. Then, slides were washed again 3x 5 minutes with PBS, secondary or primary conjugated antibodies were added (**Table 1**) and incubated for 1 h in a humid cell at 37°C. Secondary antibodies used were goat-anti-rabbit-Alexa Fluor 488 (catalog n. A-11008, Invitrogen, Carlsbad, CA, USA) and goat-anti-mouse IgG1-Alexa Fluor 647 (catalog n. A-21240, Invitrogen, Carlsbad, CA, USA). For monoclonal antibody stainings, purified mouse IgG1 was used as an isotype control. For the polyclonal antibody stainings, control stainings were performed using only secondary antibody. Finally, slides were counterstained with Hoechst (10 µg/ml) for 10 minutes and mounted on a microscope slide in mounting solution (Dabco). Images were taken with a confocal microscope (TCS SPE DM2500, Leica) using a Leica 63x/1,3 NA oil lens and processed using Fiji (25).

**Table 1:** Primary antibodies for immunofluorescence staining.

Target	Clone	Dilution	Conjugation	Supplier	Catalog #
NKp46	VIV-KM1	1/100	Unconjugated	Invitrogen	MA5-28353
CD31/PECAM-1	Rabbit Polyclonal	1/100	Unconjugated	NovusBio	NB100-2284
pan-Cytokeratin	C-11	1/100	Alexa Fluor^®^ 594	BioLegend	628606

### RNA-seq analysis

Three porcine NK cell populations (circulating blood CD8α^high^ NK cells, liver CD8α^dim^ NK cells and liver CD8α^high^ NK cells) of five six-months-old pigs were FACS-purified. The total number of cells collected for each NK cell subpopulation and each animal ranged between 1.1 million and 2 million cells. Cells were lysed in RLT buffer and total RNA isolation was performed using the RNeasy mini kit (catalog n. 74004, Qiagen, Venlo, The Netherlands). The QuantSeq 3’ mRNA-seq library prep kit (Lexogen, Vienna, Austria) was used to sequence mRNA from the 3’ end on a Nextseq500 (Illumina, San Diego, CA, USA). FastQ files were trimmed using the QuasR package (26), followed by alignment using Rhisat2 (23) to the *Sus scrofa* reference genome version 11.1. Quality metrics were computed using FastQC. The R-package DESeq2 (24) was used to identify differentially expressed genes between each group (n=5). Gene set enrichment was performed using Enricher from the Clusterprofiler package (27) on differentially expressed genes with an adjusted p-value of <0.05 and a BaseMean >500. Specific pathway information was visualized using the Pathview package (28). In all of the analyses, all three NK cell subpopulations of all five data points (individual pigs) were included.

### Porcine blood NK cell culture

For the plasticity experiments, porcine blood NK cells were cultured in 96-well flat-bottomed plates (Nunc, Thermo Fisher Scientific) at a density of 2.5 × 10^6^ cells/ml in 200 µl RPMI (Gibco, Thermo Fisher Scientific, Waltham, MA, USA), supplemented with 10% (v/v) fetal calf serum (Thermo Fisher Scientific, Waltham, MA, USA), 100 U/ml penicillin (Gibco, Thermo Fisher Scientific, Waltham, MA, USA), 100 μg/ml streptomycin (Gibco, Thermo Fisher Scientific, Waltham, MA, USA) (referred to as porcine NK medium). NK cells were primed with recombinant human interleukin 2 (IL-2) (20 ng/ml; catalog n. PHC0026, Thermo Fisher Scientific, Waltham, MA, USA) or recombinant porcine IL-10 (30 ng/ml; catalog n. 693 PI, R&D systems, Minneapolis, MN, USA) or a cytokine mix containing recombinant human TGF-β1 (10 ng/ml; catalog n. 7754-BH-005, R&D systems, Minneapolis, MN, USA) and IL-10 (30 ng/ml) or TGF-β1 (10 ng/ml) and IL-2 (20 ng/ml) for 60 h with a medium change after 40 h. Viability of fresh porcine NK cells was > 90%, wereas viability after 60 h in cell culture ranged between 55-65% for conditions without IL-2 (IL-10 and TGF-β + IL-10) and between 70-80% for conditions with IL-2 (IL-2 and TGF-β + IL-2). 

### Flow cytometry

#### Cell surface marker staining

Freshly isolated NK cells were stored overnight at 4°C in porcine NK medium with 25% fetal calf serum (FCS) and cultured NK cells were collected from the 96-well plates after 60 h of incubation before analysis. NK cells were washed in PBS-EDTA with 1% FCS (further referred to as staining medium) and incubated for 30 minutes with 0.1% Fixable Live/Dead stain (catalog n. L34963, Invitrogen, Carlsbad, CA, USA). Cells were transferred to 96-well conical bottomed plates and incubated for 30 minutes at 4°C with primary antibodies or isotype as listed in **Table 2**. After incubation, cells were washed, stained with goat anti-mouse IgG1-PE (catalog n. P21129, Invitrogen, Carlsbad, CA, USA) for 30 minutes at 4°C. Cells were washed again and resuspended in 100 µl staining medium for analysis. Flow cytometry was performed using a Beckman Coulter Cytoflex and samples were analyzed using CytExpert software (Beckman Coulter, Brea, CA, USA).

**Table 2:** Primary antibodies for flow cytometry.

Target	Clone	Conjugation	Supplier	Catalog #
NKp46	VIV-KM1	Unconjugated	Invitrogen	MA5-28353
CD49e	VC5	Unconjugated	BD biosciences	555651
CD8α	11/295/33	Unconjugated	In-house production	–
CD3	PPT3	Unconjugated	In-house production	–
Eomes	WD1928	PE	Invitrogen	12-4877-42
T-bet	4B10	PE	Invitrogen	12-5825-82

# = number.

- = Not applicable.

#### Transcription factor staining

Freshly isolated NK cells were stored overnight at 4°C in porcine NK medium with 25% FCS and cultured NK cells were collected from the 96-well plates after 60 h of incubation before analysis. NK cells were washed in PBS-EDTA with 1% FCS (staining medium) and incubated for 30 minutes with 0.1% Fixable Live/Dead stain (catalog n. L34963, Invitrogen, Carlsbad, CA, USA). Cells were transferred to 96-well conical bottomed plates and fixed and permeabilized using the Foxp3/transcription factor staining buffer set (catalog n. 00-5523-00, Thermo Fisher, Waltham, MA, USA), according to the manufacturer’s instructions. Next, cells were incubated with fluorescently labeled antibodies against the corresponding transcription factors or isotype control antibodies (**Table 2**) at 4°C for 30 min and washed with permeabilization buffer (catalog n., 00-5523-00, Thermo Fisher, Waltham, MA, USA). Finally, cells were resuspended in 100 µl staining medium for analysis. Flow cytometry was performed using a Beckman Coulter Cytoflex, and samples were analyzed using CytExpert software (Beckman Coulter, Brea, CA, USA).

### Statistical analysis

Statistical analysis of plasticity data was performed using Graphpad Prism 9. Data were analyzed for statistical differences with a Kruskal-Wallis test at the 5% significance level. *Post hoc* analysis was performed using Dunn’s multiple comparisons test.

## Results

### Porcine liver-resident CD8α^dim^ NK cells are located in the liver sinusoids and display a distinct transcriptomic profile compared to circulating blood NK cells and CD8α^high^ liver NK cells

**Figure 1:**
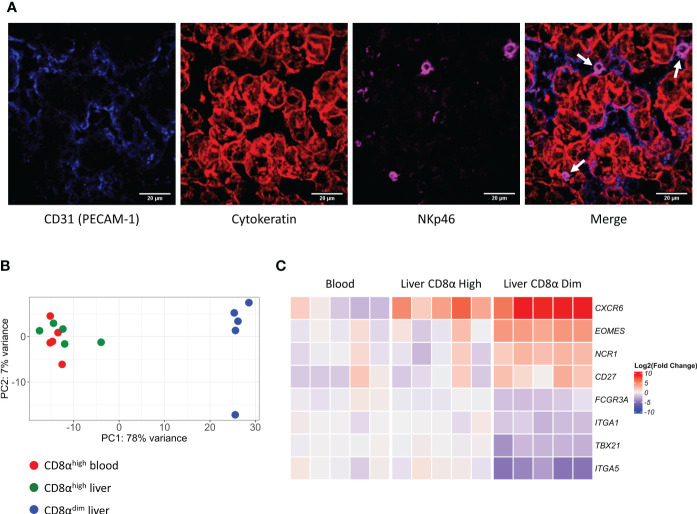
Liver-resident CD8α^dim^ NK cells are located in the liver sinusoids and show a distinct transcriptomic profile compared to liver CD8α^high^ NK cells and circulating blood CD8α^high^ NK cells. **(A)** Immunofluorescence staining of liver tissue. NK cells (white arrows) expressing NKp46 (in magenta) are located in the sinusoids, in close contact with the endothelium (PECAM-1, in blue). Hepatocytes stain positive for pan-Cytokeratin (in red). **(B)** Principal component analysis (PCA) of the transcriptomes of CD8α^high^ blood NK cells, CD8α^high^ liver NK cells and CD8α^dim^ liver-resident NK cells. **(C)** Heatmap of gene transcripts of the three described NK cell subsets, displaying the transcription levels of proteins that were previously used to phenotypically characterize liver-resident NK cells.

We have previously shown that lrNK cells are abundantly present in the liver of six-months-old pigs (45.5 ± 13.1% of the lymphocytes) compared to the presence of their circulating counterparts in the blood of these pigs at the same age (21 ± 5.2% of the lymphocytes) (21). Since murine and human lrNK cells were described to reside in liver sinusoids (12, 29), we were interested to know whether this was also the case for porcine lrNK cells. To identify a suitable marker to specifically stain lrNK cells on tissue sections for microscopy, lymphocytes obtained from a flushed liver were phenotypically characterized. NKp46 was found to be expressed by all CD8α^dim^ lrNK cells, whereas only ∼3% of the NKp46-expressing cells did not belong to the CD8α^dim^ NK cell population.This small percentage of non-lrNK cells expressing NKp46 were characterized as CD8α^high^CD3^+^ lymphocytes, which might correspond to a rare NKp46-expressing T cell subset and/or a non-conventional lymphocyte subset that was previously described to be enriched in the porcine liver and that displays both T cell and NK cell features, but functionally resembles NK cells (30). In contrast, CD8α^high^ NK cells in the liver did not express detectable levels of NKp46. Therefore, NKp46 was chosen as a suitable marker for CD8α^dim^ lrNK cells (**Supplementary Figure 3**). As shown in **Figure 1A**, porcine lrNK cells reside in the liver sinusoids, in close contact with the CD31-expressing sinusoidal endothelium. This is in line with their human and mouse counterparts.

To further characterize and compare porcine NK cell subsets, we isolated CD8α^high^ conventional peripheral blood NK cells, CD8α^high^ conventional liver NK cells and CD8α^dim^ liver-resident NK cells from five different animals and performed an RNA-seq analysis on these samples (an overview of the differentially expressed genes (DEGs) can be found in **Supplementary Tables 1–3**). As visualized by the principal component analysis (PCA) (**Figure 1B**), CD8α^high^ conventional blood NK cells and CD8α^high^ conventional liver NK cells cluster together on transcriptional level, whereas the CD8α^dim^ liver-resident NK cells cluster apart from both populations.

We previously described and phenotypically characterized the CD8α^dim^ lrNK cell population by flow cytometry (21). Hence, we compared the transcriptome dataset with this phenotypic characterization on the protein level to determine whether differences in protein levels are reflected by similar differences on transcript level. Interestingly, in line with our previous observations at the protein level, we found that CD8α^dim^ lrNK cells display a significantly increased expression of *EOMES* mRNA and a significant decrease in *TBX21* transcripts, compared to conventional CD8α^high^ blood NK cells. In addition, the low expression of *ITGA5* (encoding CD49e) transcripts and the high expression levels of *CXCR6*, *NCR1* (encoding NKp46) and *CD27* mRNA (**Figure 1C**) are further in line with our earlier flow cytometric characterization (21), thereby validating both the earlier and the current report. Furthermore, in line with human lrNK cells and in contrast with murine lrNK cells (9, 16), porcine CD8α^dim^ lrNK cells did not show high expression of *ITGA1* (encoding CD49a).

### CD8α^dim^ lrNK cells display an immature and anti-inflammatory phenotype

**Figure 2:**
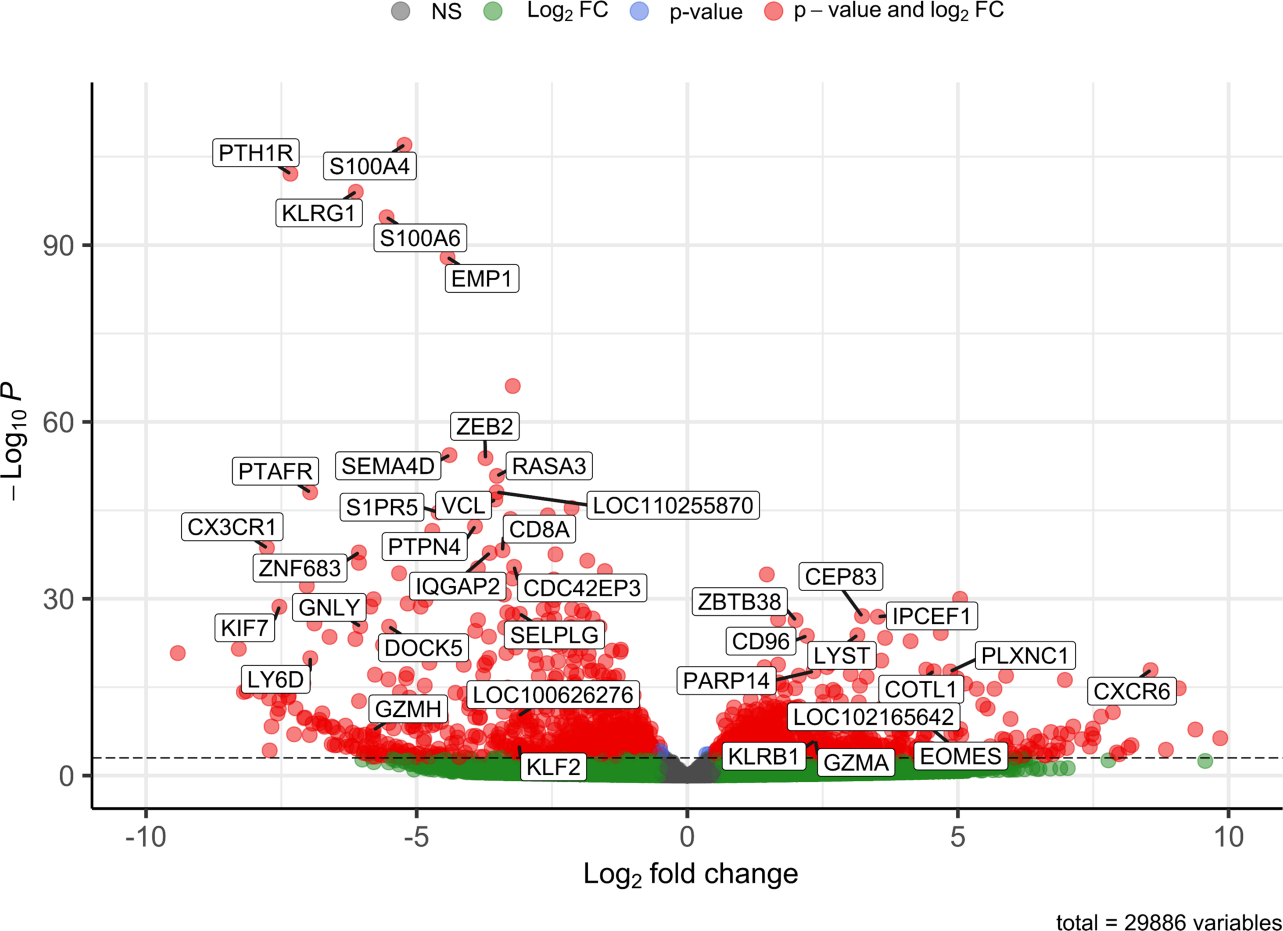
Volcano plot visualizing 39 differentially expressed genes between CD8α^dim^ liver-resident NK cells and conventional blood NK cells. Only transcripts with a BaseMean > 500 and a significant Log2 fold change higher than 2 or lower than -3 are labeled.

In order to obtain a general overview of the most pronounced transcriptional differences between the CD8α^dim^ lrNK cell population and conventional blood NK cells, we first analyzed the genes that were abundantly expressed (BaseMean > 500) and that showed a significant Log2 fold change higher than 2 or lower than -3 (since in general more genes showed a negative fold change) (**Supplementary Table 1**). Using these criteria, 39 differentially expressed genes were identified between the CD8α^dim^ lrNK cell population and conventional blood NK cells (**Figure 2**).

A presumably anti-inflammatory function of CD8α^dim^ lrNK cells was suggested by a high expression of *CD96* (encoding the activating receptor Tactile) and by a low expression of *SEMA4D* (encoding CD100, Semaphorin-4D), encoding an immune activation molecule, and of *GNLY* (Granulysin), encoding a powerful cytotoxic, antimicrobial and pro-inflammatory peptide (31–34). However, abundant expression of genes associated with lytic granules, such as *LYST* (encoding lysosomal trafficking modulator) and *GZMA* (encoding granzyme A), and *PARP14*, encoding a critically important molecule for IFN-γ production, indicate that these cells do have the ability to exert cytotoxic functions and produce cytokines upon activation (35, 36). Nevertheless, granzyme A might preferably act as an immunomodulatory molecule in the pig liver, since human granzyme A has recently been described as a modulator of inflammation rather than a cytotoxic molecule (37).

In addition, *CXCR6* transcripts are highly expressed in the CD8α^dim^ lrNK cell population. The ligand for this receptor, CXCL16, is constitutively expressed in the liver and acts as a chemoattractant for CXCR6-expressing cells. Therefore, CXCR6 is regarded as a marker for tissue-resident cells. Besides the high expression of the tissue-resident marker CXCR6, CD8α^dim^ lrNK cells also showed a very low expression of *CX3CR1*, a marker for circulatory leukocytes, further indicating a tissue-resident phenotype for these cells (38).

Several genes involved in F-actin binding and remodeling of the cytoskeleton, such as *S100A4*, *S100A6*, *PLXNC1* and *COTL1* (39–42), were also differentially expressed. For example, expression of *S100A4* and *S100A6* was strongly reduced in CD8α^dim^ lrNK cells. In contrast, these cells showed a high expression of *PLXNC1* and *COTL1*. Interestingly, *COTL1* is known to be induced by TGF-β (43), which was recently reported to cause downregulation of *ZEB2*, *S1PR5* and *KLF2* in human and murine T cells and NK cells to enforce tissue retention of these cells (44). In line with this, we also noticed a downregulation of these three TGF-β sensitive genes, implying that also in the pig liver, TGF-β is a driving force for tissue retention of lrNK cells, mediated *via* downregulation of *S1PR5* in these cells. Moreover, *ZEB2* and *S1PR5* are markers of NK cell maturation, thus low expression of these transcripts indicates a more immature phenotype (45, 46). Also *KLRG1*, another maturation marker in NK cells (47), was only very weakly expressed by CD8α^dim^ lrNK cells. A low expression of *KLRG1* in NK cells is associated with a higher survival and proliferative potential (48, 49). Altogether, these data indicate that lrNK cells show an immature and anti-inflammatory phenotype, but possibly can still react to pathogens when triggered by specific stimuli.

### CD8α^dim^ liver NK cells highly express genes associated with the (adaptive) immune response and IFN-γ signaling, whereas lowly expressed genes are associated with cell migration and extravasation

**Figure 3:**
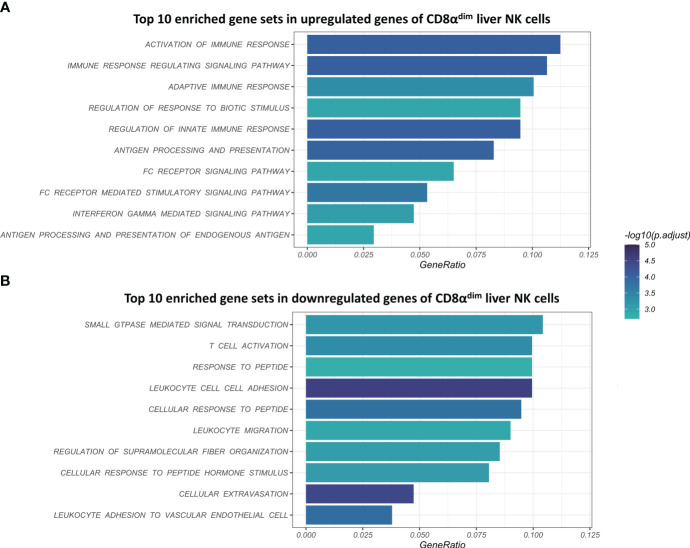
Visualization of the top 10 enriched gene sets of CD8α^dim^ liver-resident NK cells compared to CD8α^high^ conventional blood NK cells upon gene set analysis (GSEA) based on gene ontology (GO). **(A)** Top 10 enriched gene sets in upregulated genes of CD8α^dim^ liver NK cells. **(B)** Top 10 enriched gene sets in downregulated genes of CD8α^dim^ liver NK cells.

Using gene-set enrichment analysis (GSEA) based on gene ontology (GO), we found that CD8α^dim^ lrNK cells showed a significantly higher expression of gene sets such as “activation of immune response”, “adaptive immune response”, “antigen processing and presentation”, “antigen processing and presentation of peptide antigen” and “interferon gamma mediated signaling pathway” (**Figure 3A**). In general, these highly expressed gene sets are linked to the regulation of the (adaptive) immune response and IFN-γ signaling. Interestingly, we found that the two porcine MHC II transcripts (*SLA-DQ* and *SLA-DR*) showed a significantly higher expression in CD8α^dim^ lrNK cells compared to cNK cells. This might point towards an antigen-presenting function of these cells, since it has been shown that porcine NK cells can indeed upregulate SLA-DR upon vaccination and may process and present antigens *via* MHC II to T cells (22, 50). Also transcripts encoding proteins that are involved in an efficient IFN-γ response, such as *PARP14*, *JAK1* and *STAT1* were more abundantly expressed in CD8α^dim^ lrNK cells compared to cNK cells. On the other hand, CD8α^dim^ lrNK cells showed low expression of transcripts associated with leukocyte migration and extravasation in comparison to cNK cells (**Figure 3B**). For example, *CX_3_CR1*, *ITGA1*, *ITGA4*, *ITGAL*, *ITGB2*, *ITGB7*, *CD44*, *SPN* and *MSN* transcripts were significantly less expressed, highlighting again the resident nature of these cells.

**Figure 4:**
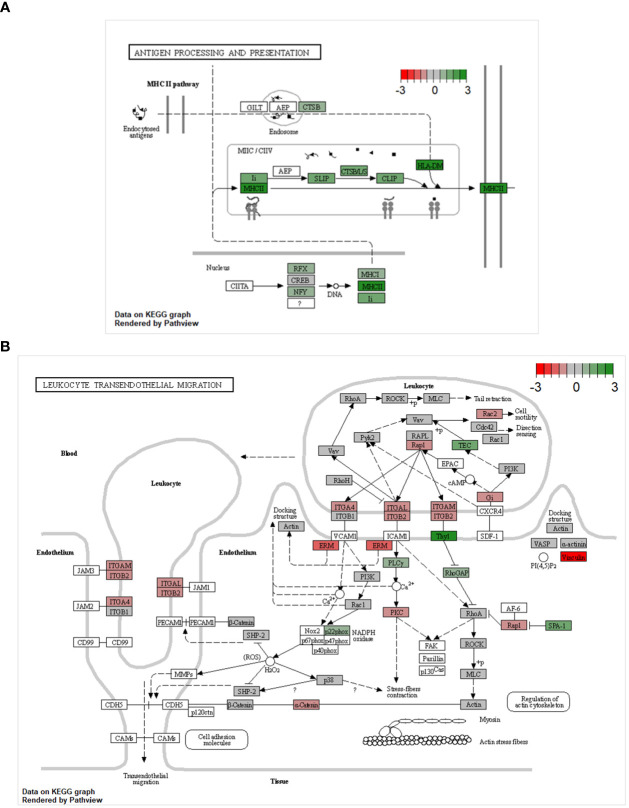
KEGG pathway analysis comparing the transcriptome of CD8α^dim^ liver-resident NK cells to the transcriptome of conventional blood NK cells. KEGG pathways showing transcripts upregulated in CD8α^dim^ liver-resident NK cells compared to conventional blood NK cells in green and downregulated transcripts in red **(A)** KEGG antigen processing and presentation pathway. Transcripts of several proteins involved in the MHC II pathway are more abundantly expressed in lrNK cells compared to cNK cells. **(B)** KEGG leukocyte transendothelial migration pathway. All the detected surface proteins involved in trans-endothelial migration, except for ITGB1, are expressed to a much lower extent in lrNK cells compared to cNK cells.

The GSEA pointed towards major changes in the trans-endothelial migration and antigen-presenting capacities of CD8α^dim^ lrNK cells compared to blood NK cells. Therefore, we used Kyoto Encyclopedia of Genes and Genomes (KEGG) pathway analysis to visualize the complete pathways of both processes. KEGG pathways are used to link genomic information with functional cellular processes based on the current knowledge. **Figure 4** shows KEGG pathways with transcripts upregulated in CD8α^dim^ lrNK cells compared to cNK cells in green, and downregulated transcripts in red. Regarding antigen processing and presentation, we could confirm that transcripts of several proteins involved in the MHC II pathway were more abundantly expressed in lrNK cells compared to cNK cells (**Figure 4A**), indicated that this pathway is probably functionally enhanced in lrNK cells. For leukocyte trans-endothelial migration, we observed that all the detected surface proteins involved in trans-endothelial migration, except for ITGB1, were expressed to a much lower extent in CD8α^dim^ lrNK cells compared to cNK cells (**Figure 4B**), which indicates a less migratory phenotype of these cells, thereby implying a more tissue-resident nature.

### CD8α^high^ liver NK cells represent a putative intermediate cell population expressing transcripts associated with conventional blood NK cells as well as with liver resident NK cells

**Figure 5:**
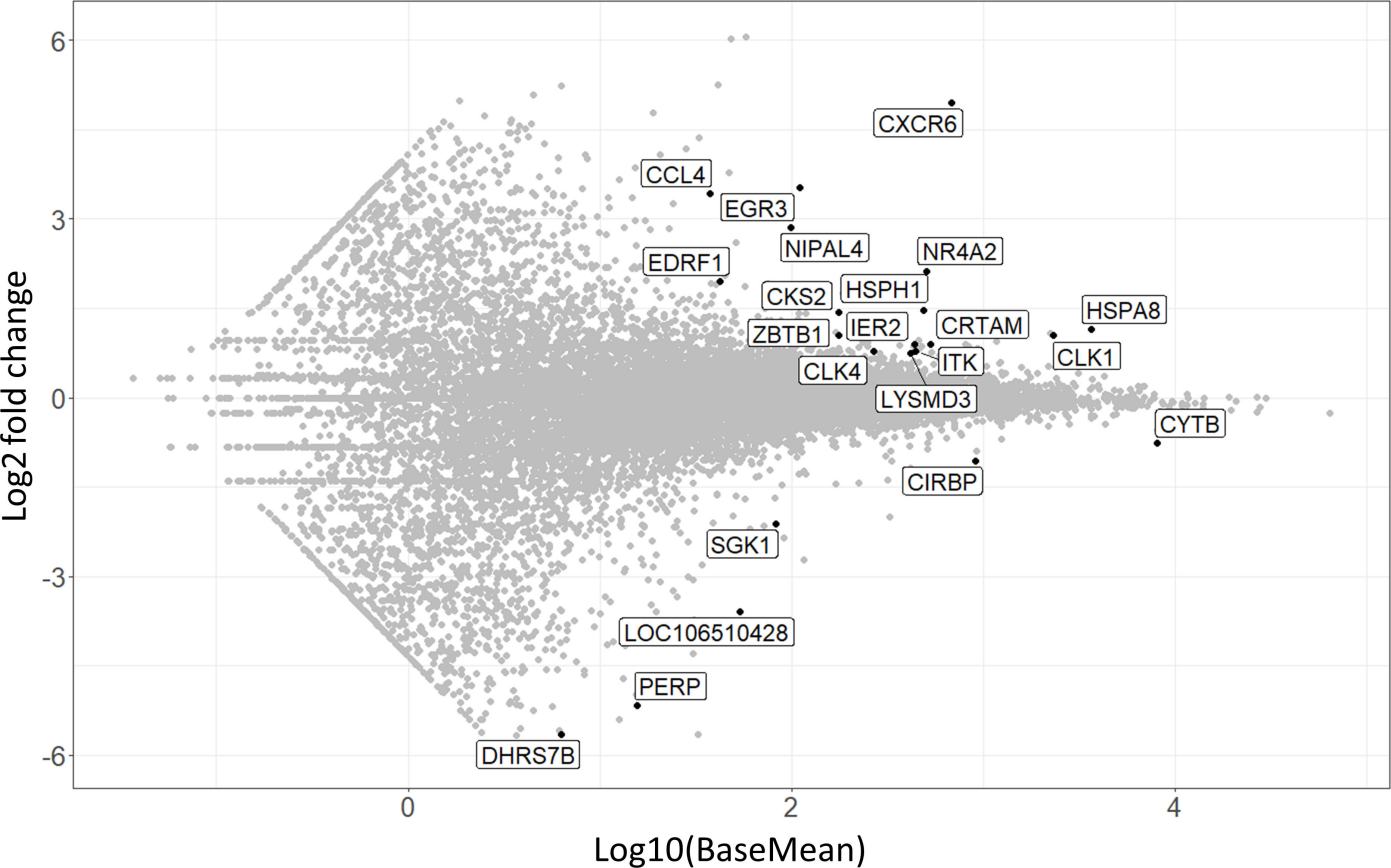
MA-plot visualizing the 22 differentially expressed transcripts in CD8α^high^ liver NK cells compared to conventional peripheral blood NK cells. The x-axis shows expression levels, log_2_ fold change is shown on the y-axis.

Besides analyzing the CD8α^dim^ lrNK cell population, we were also interested to see whether the CD8α^high^ liver NK cell population was either a passer-by population of cNK cells, accidently present in the liver at the moment of liver collection, or rather an intermediate cell population either ‘preparing’ for liver residence or just exiting the liver parenchyma. The former explanation was deemed less likely since the liver was flushed with 5 l ice-cold PBS before collecting the cells, thereby likely removing the vast majority of circulating immune cells. We observed that this CD8α^high^ liver NK cell population transcriptionally strongly resembles cNK cells isolated from the peripheral blood, although 22 transcripts were found to be significantly differentially expressed between CD8α^high^ liver NK cells and cNK cells (**Figure 5**) (**Supplementary Table 2**).

Interestingly, *CXCR6* was among these 22 transcripts, and showed the highest fold change of all transcripts. This indicates that CD8α^high^ liver NK cells isolated from the liver actively upregulate *CXCR6* transcription. Intriguingly, these high levels of *CXCR6* transcripts do not immediately lead to increased CXCR6 expression at the protein level on the surface of these cells, as we could not detect an increased CXCR6 protein expression on CD8α^high^ liver NK cells by flow cytometry (21). Other transcripts that were more abundantly expressed in CD8α^high^ liver NK cells compared to cNK cells included *EGR3*, *NR4A2* and *HSPA8*, which have all been described to be negative immune regulators (51–56). NR4A2, for example, is known as a transcription factor involved in NK cell development and NK cells that express high levels of this transcription factor had a poor cytotoxic activity against tumor cells (53).

### Indications for (partial) *in vitro* plasticity from cNK cells towards CD8α^dim^ lrNK cells

We previously reported limited plasticity between blood cNK cells and CD8α^dim^ lrNK cells, since stimulation of cNK cells with IL-2/12/18 resulted in upregulation of CXCR6 cell surface expression (21). We aimed to further elaborate on these plasticity assays based on the information obtained from this RNA-seq data set. As indicated above, the transcriptomic profile of the CD8α^dim^ lrNK cell population pointed towards a strong influence of TGF-β on these cells. Furthermore, TGF-β and IL-10 were abundantly expressed in healthy human liver (57), although only TGF-β was found to downregulate T-bet expression in human cNK cells (58). TGF-β was thus identified as a critically important regulator of the phenotype of lrNK cells in man (58). Unfortunately, to our knowledge, no studies specifically examining the expression of IL-10 or TGF-β in the pig liver have been performed thus far. The porcine liver cell atlas (59) did show IL-10 transcripts to be expressed by Kupffer cells, the liver-resident macrophages. Since this atlas focuses only on immune cells present in the pig liver, it does not allow to evaluate cytokine expression levels by parenchymal cells.

**Figure 6:**
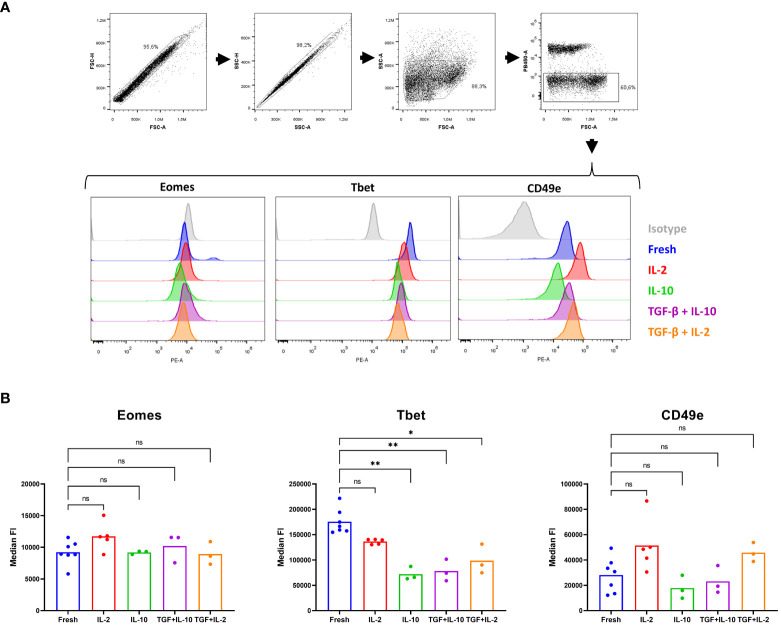
A partial in vitro plasticity is observed upon culturing of conventional blood NK cells with IL-10 and TGF-β. cNK cells were isolated from blood (n = 3 to7) and cultured in the presence of IL-2, IL-10 or combined with TGF-β for 60 h. Subsequently, in freshly isolated cNK cells or cultured cNK cells, expression of three lrNK cell markers were evaluated by flow cytometry. **(A)** Gating strategy upon staining of fresh or cultured isolated cNK cells. Doublets were excluded based on their forward scatter and side scatter pattern. The light scatter properties of the sorted NK cells were verified. Dead cells were excluded based on a live/dead marker. Histograms show the expression of Eomes, Tbet, CD49e and isotype control for the different conditions. **(B)** Median fluorescence intensity (FI) of live cells, determined in the PE channel (Eomes, Tbet and CD49e, respectively), is shown. *p < 0.05; **p < 0.01; ns, not significant.

Therefore, we decided to investigate the effect of TGF-β and IL-10 on porcine cNK cells. In human, plasticity experiments over multiple days are performed in a basal medium containing rhIL-15 to assure NK cell viability (60). In pig, however, rpIL-15 is not sufficient to retain satisfying viability in NK cells and TGF-β treatment was therefore combined with either IL-2 or IL-10, cytokines that are able to maintain porcine cNK cell viability (61, 62). In line with a report in human (58), IL-10 nor TGF-β were able to increase Eomes expression in porcine cNK cells, but TGF-β was able to downregulate T-bet expression in these cells. In contrast to human (58), porcine NK cells also significantly downregulated T-bet expression when treated with IL-10. Furthermore, a trend towards downregulated expression of the integrin CD49e was also observed upon IL-10 treatment of cNK cells, although this difference did not reach statistical significance (**Figure 6**). These results further support the idea that (partial) plasticity from cNK cells towards lrNK cells may exist in pig, and that TGF-β and IL-10 likely contribute to such process, although on their own they are not sufficient to induce complete transition from cNK cells to lrNK cells.

Unfortunately, we could not perform plasticity experiments with CD8α^dim^ lrNK cells to assess their transition towards a cNK cell phenotype, since their viability is severely compromised upon cultivation. Neither IL-2, IL-10, IL-15 nor TGF-β or combinations of these cytokines were able to maintain satisfactory viability of lrNK cells (data not shown).

## Discussion

Liver-resident NK cells, as well as other tissue-resident NK cell populations were found to display features that are usually not linked to the innate immune system, such as memory-like responses and longevity (6, 15, 63). Whereas substantial differences exist between lrNK cell populations in mice and human, we have recently identified a porcine CD8α^dim^ lrNK cell population that strongly resembles human lrNK cells (21). In this report, we further characterize this porcine lrNK cell population and compare its transcriptome to that of cNK cells from peripheral blood. Furthermore, we compared both NK cell populations with a second CD8α^high^ NK cell population in the pig liver.

We revealed that, in line with data obtained in man, porcine CD8α^dim^ lrNK cells generally display an immature and anti-inflammatory transcriptome. The liver is generally considered as a tolerogenic environment and thus NK cells residing in this milieu seem to adapt to its anti-inflammatory status. In human, TGF-β is thought to play a crucial role in this process (58), and in pig we found that several of the differentially expressed genes between cNK cells and lrNK cells were controlled by TGF-β as well. Moreover, TGF-β was able to downregulate T-bet expression of porcine cNK cells *in vitro*, indicating a possible plasticity of NK cells between the two compartments.

When studying tissue-resident NK cells, it is important to be aware that another poorly cytotoxic innate immune cell population, highly resembling NK cells, and residing in tissues such as the liver has been described, namely type 1 innate lymphoid cells (ILC1). Due to overlapping phenotypes in the liver, a clear distinction between lrNK cells and ILC1 is controversial, especially in human (64, 65). In contrast, in mice, a liver-specific ILC1 population was identified and is characterized by the expression of CD49b and CD200R1 (66). TGF-β was shown to be involved in the conversion of NK cells towards a less cytotoxic ILC1 population (67, 68). In pig, CD8α^dim^ lrNK cells do not express CD49b or CD200R1, but share the poorly cytotoxic phenotype of murine ILC1. However, like in human, there is no clear evidence for a separate ILC1 population in the pig liver. This is in line with a recently reported liver cell atlas based on single-cell RNA sequencing of livers from different species, where no separate cluster of ILC1 was found in human or pig, whereas in mice a separate ILC1 cluster was identified (59).

In contrast to human (58), in pig, IL-10 also seems to be able to influence T-bet levels in cNK cells, although the biological significance of this cytokine in shaping lrNK cells is currently unclear. Moreover, a cytokine mix containing TGF-β and IL-10 was not sufficient to maintain the viability of porcine lrNK cells *in vitro*. Additional and currently unknown factor(s) are thus needed for survival and potentially also plasticity of porcine lrNK cells. Whether such factors include chemokines, like for example the CXCR6 ligand CXCL16, and/or survival signal(s) that may depend on cell-cell interactions of lrNK cells with other hepatic cell types is currently unclear and needs further investigation.

Although their origin and development remain elusive, the current transcriptome study allows us to begin to understand the biological functions of lrNK cells in pig. We observed an upregulation of genes associated with antigen presentation, including MHC class II. This indicates that CD8α^dim^ lrNK cells possess immunomodulatory capacities and that it may be warranted to assess whether they may serve as antigen-presenting cells or tissue-resident memory NK cells, as was described previously in mice (6, 63) and human (15). Possibly in line with a potential role in antigen presentation, we previously reported that porcine peripheral blood NK cells can express MHC class II and costimulatory molecules, internalize antigens derived from killed target cells, and may stimulate T cell proliferation (22, 50). Together, these data point towards a very broad and versatile functional repertoire of porcine NK cells. Whether porcine CD8α^dim^ lrNK cells do indeed function in adaptive immune responses – either as antigen-presenting cells, tissue-resident memory NK cells or other – *in vivo* is still unknown and will likely require vaccination and/or infection trials.

Finally, the CD8α^high^ liver NK cell population we characterized in this report, appears to represent an intermediate NK cell population that transcriptionally closely resembles blood cNK cells, but also shows expression of particular transcripts that are associated with the CD8α^dim^ lrNK cell population. This is, to the best of our knowledge, the first description of such a putative intermediate population of liver NK cells in any species and might contribute to further elucidate the origin and development of the lrNK cell population. These CD8α^high^ NK cells upregulate transcription of some tissue-resident markers, such as CXCR6, and adopt a more anti-inflammatory transcriptome, seemingly preparing for liver residency. We speculate that under steady-state conditions, these cells may exit the liver again and lose this preparatory phenotype, while, upon encountering specific stimuli, they might adopt a truly tissue-resident phenotype and become tissue-resident cells.

Taken together, we showed that lrNK cells in the pig express high levels of genes related to antigen presentation and generally display an anti-inflammatory and immature phenotype. This confirms that lrNK cells are valuable novel targets for future vaccination strategies or novel therapies against liver-associated diseases. Furthermore, we suggest that, as in human, TGF-β is important for plasticity between blood and liver NK cell subsets, although IL-10 might provide additional support in pig. We also characterized the CD8α^high^ liver NK cell population as a putative intermediate subset that might be exploited to provide additional information regarding plasticity in the future. Porcine lrNK cells display poor survival *in vitro*, even when supplementing with different cytokines, indicating that one or more cardinal factor(s) in porcine lrNK viability and cell biology remain elusive and need to be determined in the future. Although the CXCL16-CXCR6 signaling axis has been described in many studies as a potentially important survival factor for CXCR6-expressing cells (15, 69), we could not enhance the viability of porcine lrNK cells by adding porcine recombinant CXCL16 to the culture medium (data not shown). Potentially additional chemokines might be involved, since our current transcriptome study shows that lrNK cells display increased expression of e.g. transcripts encoding CCR6 and CCR9. In addition, cell-cell interactions might be crucial for lrNK cells in order to survive. Therefore, we believe that establishing live organoids in which CXCL16 and potential other relevant factors can be expressed by sinusoidal cells as is the case *in vivo*, might be a promising future step to further unravel the functional and biological role of lrNK cells. Such studies will allow to further address the value of the pig as an animal model to translate features of lrNK cells into innovative clinical applications, both in animal and man.

## Data availability statement

The datasets generated and analyzed for this study can be found in NCBI’s Gene Expression Omnibus (GEO) repository and are accessible through GEO Series accession number GSE229497 (https://www.ncbi.nlm.nih.gov/geo/query/acc.cgi?acc=GSE229497).

## Ethics statement

The animal study was reviewed and approved by Ethical Committee of the Faculty of Veterinary Medicine, Ghent University (EC2017/121).

## Author contributions

LH, SDP, SD, and EH-S performed the experiments. LH, EM, EC, HF, and BD designed the research and wrote the manuscript. LH and RJ made the figures. LH, RJ, DD, and FV analyzed the results. All authors contributed to the article and approved the submitted version.
