# Supplementary material for: Comparative transcriptomics of porcine liver-resident CD8αdim, liver CD8αhigh and circulating blood CD8αhigh NK cells reveals an intermediate phenotype of liver CD8αhigh NK cells
Source: Front Immunol. 2023 Aug 18;14:1219078. doi: 10.3389/fimmu.2023.1219078 (PMC10471975; doi:10.3389/fimmu.2023.1219078)
Supplement: Supplementary file 1 [file DataSheet_1.docx]

Supplementary Material

Comparative Transcriptomics of Porcine Liver-Resident CD8α^dim^, Liver CD8α^high^ and Circulating Blood CD8α^high^ NK cells Reveals an Intermediate Phenotype of Liver CD8α^high^ NK cells

# Supplementary figures

##
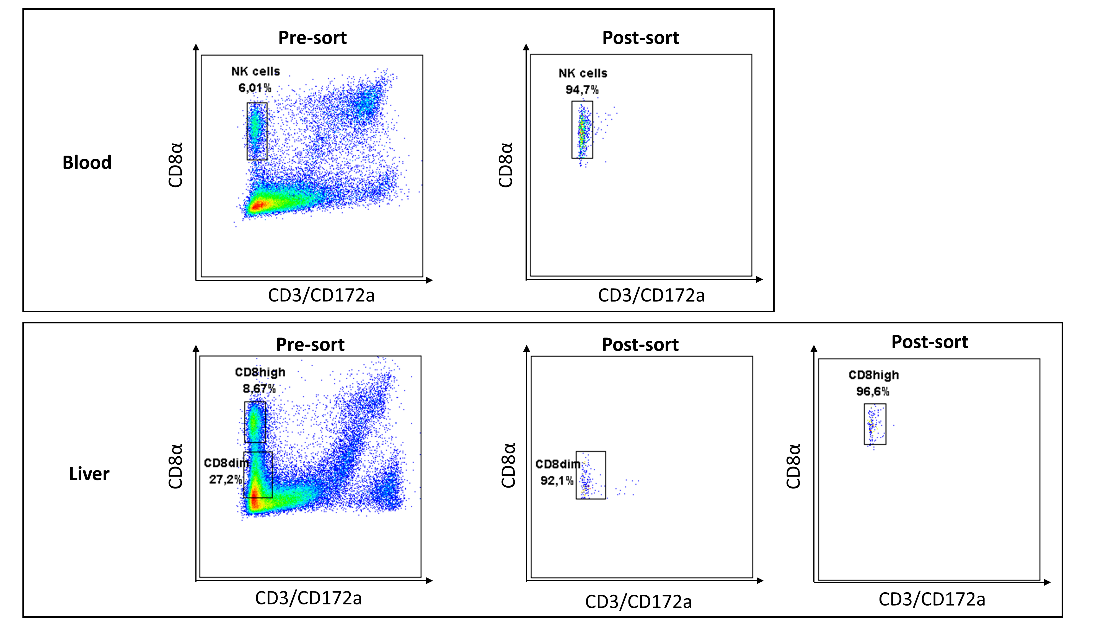
Supplementary figure 1

**Supp. Figure 1: Pre- and post-sort analysis of porcine blood and liver NK cells.**

## Supplementary Figure 2

**Supp. Figure
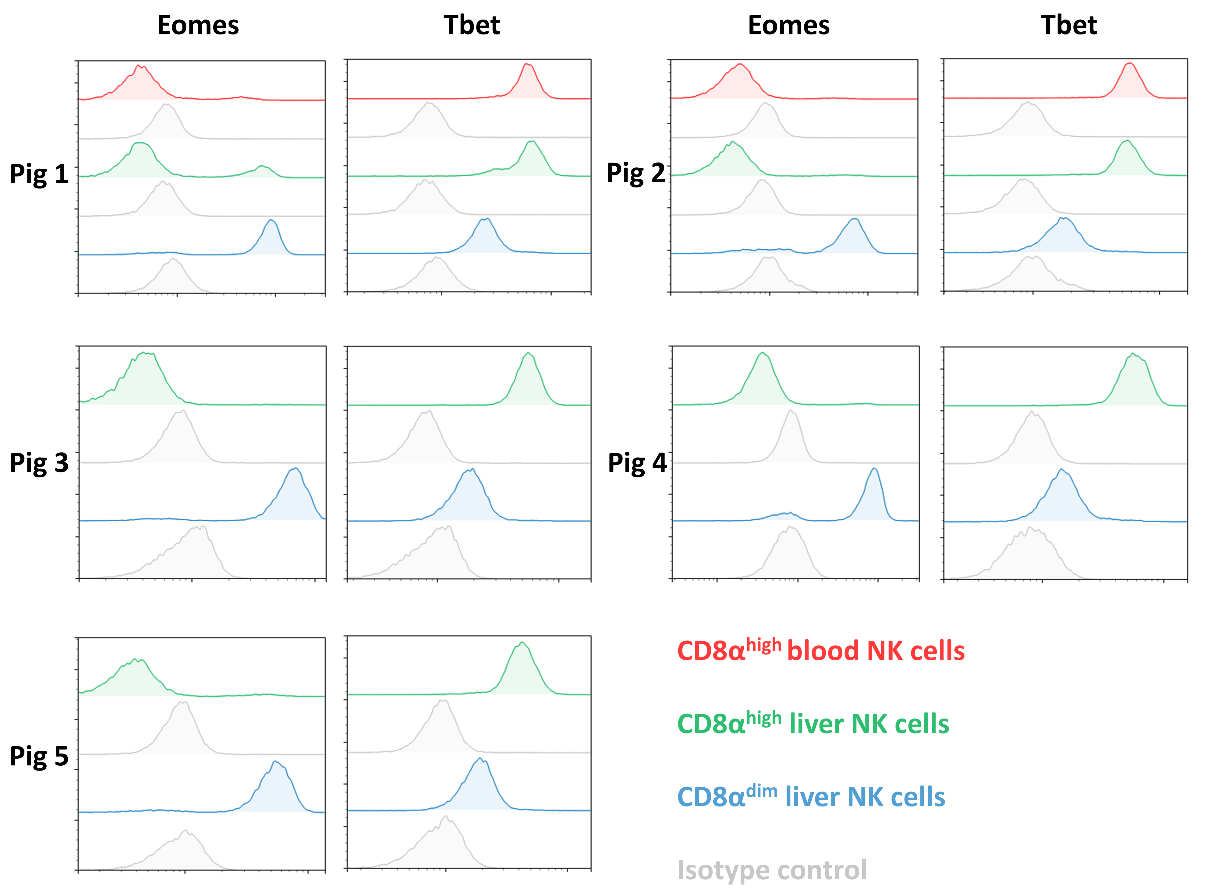
2: Flow cytometric histograms showing the expression of Eomes and Tbet on the sorted CD8α^high^ blood NK cells (red), CD8α^high^ liver NK cells (green) and CD8α^dim^ liver NK cells (blue).** Isotype controls (grey) are shown for each marker. Graphs show the median fluorescence intensity values for both markers. These stainings were performed to verify the purity of the sorted NK cell populations before RNA sequencing was carried out.

##
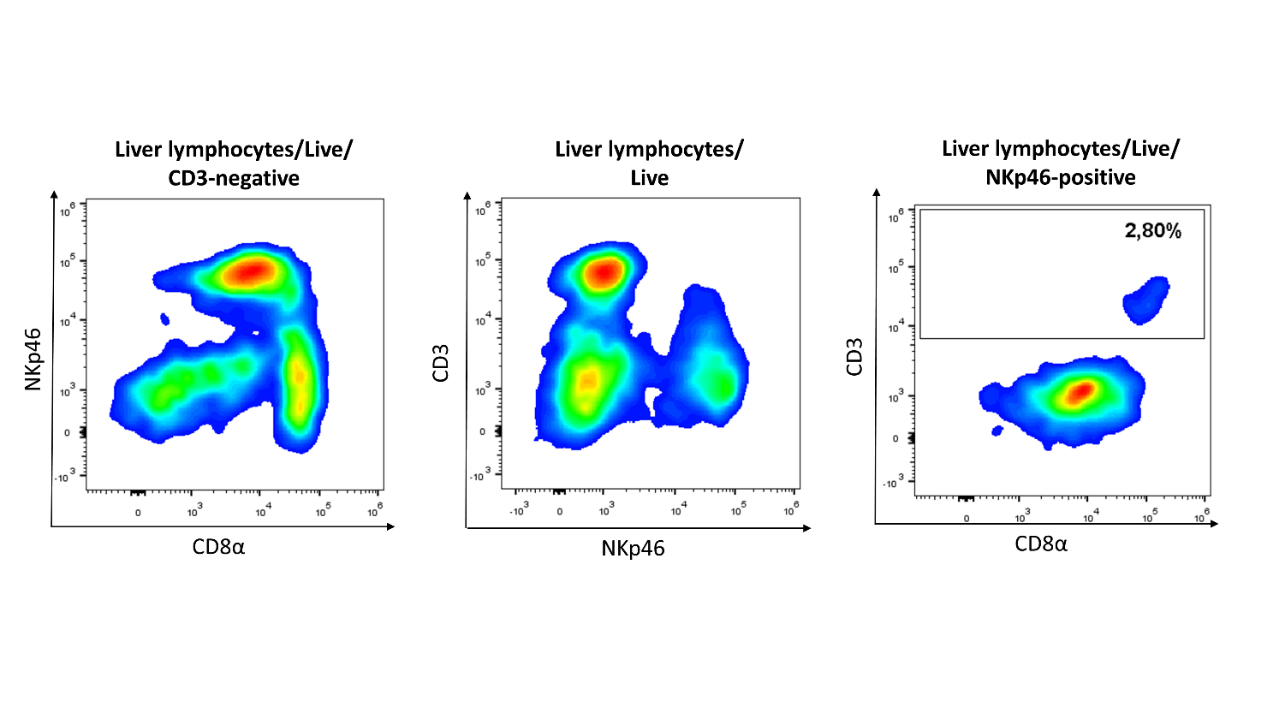
Supplementary figure 3

**Supp. Figure 3: Expression of NKp46, CD3 and CD8 by liver lymphocytes.** All porcine CD8α^dim^ lrNK cells express NKp46, whereas CD3^-^CD8α^high^ liver lymphocytes are NKp46-negative. In the total population of porcine liver lymphocytes, NKp46 is mainly expressed by CD3-negative cells, corresponding to liver-resident NK cells. However, a small fraction of CD3^+^ cells also displays NKp46 expression in the pig liver (+- 3% of all NKp46-expressing cells in the liver), these cells were characterized as CD3^+^CD8α^high^ cytotoxic T cells. This small percentage of non-lrNK cells expressing NKp46 might correspond to a rare NKp46-expressing T cell subset and/or a non-conventional lymphocyte subset that was previously described to be enriched in the porcine liver and that displays both T cell and NK cell features, but functionally resembles NK cells.

# Supplementary tables

## Supplementary table 1

Differential expression analysis of porcine conventional blood NK cells versus porcine CD8α^dim^ liver-resident NK cells.

## Supplementary table 2

Differential expression analysis of porcine conventional blood NK cells versus porcine CD8α^high^ conventional liver NK cells.

## Supplementary table 3

Differential expression analysis of porcine CD8α^high^ conventional liver NK cells versus porcine CD8α^dim^ liver-resident NK cells.
